# A New Biotechnology Method of Bioelements’ Accumulation Monitoring in In Vitro Culture of *Agaricus bisporus*

**DOI:** 10.3390/molecules26175165

**Published:** 2021-08-26

**Authors:** Agata Krakowska, Witold Reczyński, Tomasz Krakowski, Karolina Szewczyk, Włodzimierz Opoka, Bożena Muszyńska

**Affiliations:** 1 Department of Inorganic and Analytical Chemistry, Faculty of Pharmacy, Jagiellonian University Medical College, 9 Medyczna Street, 30-688 Kraków, Poland; wlodzimierz.opoka@uj.edu.pl; 2 Department of Analytical Chemistry and Biochemistry, Faculty of Materials Science and Ceramics, AGH University of Science and Technology, Adama Mickiewicza Ave. 30, 30-059 Kraków, Poland; wreczyn@agh.edu.pl (W.R.); karoszewczyk@gmail.com (K.S.); 3 Department of Machinery Engineering and Transport, Faculty of Mechanical Engineering and Robotics, AGH University of Science and Technology, Adama Mickiewicza Ave. 30, 30-059 Kraków, Poland; krakowsk@agh.edu.pl; 4 Department of Pharmaceutical Botany, Faculty of Pharmacy, Jagiellonian University Medical College, 9 Medyczna Street, 30-688 Kraków, Poland; muchon@poczta.fm

**Keywords:** dipper, monitoring of bioelements concentrations, optimization of in vitro cultures, *Agaricus bisporus*, flame atomic absorption spectrometry

## Abstract

*Agaricus bisporus* (J.E. Lange) Imbach is one the most popular species of edible mushrooms in the world because of its taste and nutritional properties. In the research, repeatability of accumulation of bioelements and biomass yield in experimentally chosen in vitro culture medium, was confirmed. The in vitro cultures were conducted on the modified Oddoux medium enriched with bioelements (Mg, Zn, Cu, Fe). The aim of the study was to create an effective method of sampling, which enabled non-invasive monitoring of metals concentrations changes in the medium, during increase of biomass in in vitro cultures. The first, indirect method of sampling was applied. The non-invasive probe (a dipper) for in vitro culture was used; hence, the highest biomass increase and metals accumulation were gained. The method also guaranteed culture sterility. The second method, a direct one, interfered the in vitro culture conditions and growth of mycelium, and as a consequence the lower biomass increase and metals’ accumulation were observed. Few cases of contaminations of mycelium in in vitro cultures were observed. The proposed method of non-invasive sampling of the medium can be used to monitor changes in the concentrations of metals in the medium and their accumulation in the mycelium in natural environment. Changes in concentrations of the selected metals over time, determined by the method of atomic absorption spectrometry, made it possible to correlate the obtained results with the specific stages of *A. bisporus* mycelium development and to attempt to explain the mechanism of sampling metals from the liquid substrate.

## 1. Introduction

*Agaricus bisporus* (J.E. Lange) Imbach (White Mushroom) is the most frequently cultivated and consumed mushroom species in the world because of its taste, smell, and nutritional qualities. Fruiting bodies of *A. bisporus* contain numerous biologically active metabolites and bioelements. This species is a valuable source of fiber, endo- and exogenic amino acids, unsaturated fatty acid (linolenic acid, linoleic acid, and palmitic acid) and has valuable substances exhibiting antioxidant activity (ergothioneine, sterols, indole, and phenolic substances; selenium, zinc, and vitamins) [1,2,3,4,5,6,7,8,9,10]. The high concentrations of antioxidant compounds, anti-atherosclerosis, and antiphlogistic allowed to assign *A. bisporus* to a group of valuable food products. Prohealth importance of *A. bisporus* is derived from anticancer activity confirmed in curing and preventing from the recurrence of breast and prostate cancer [6]. Tyrosinase and polysaccharides from *A. bisporus* fruiting bodies are responsible for anticancer properties [11,12]. The experiments demonstrated that the extracts from *A. bisporus* inhibit activity of acetylcholinesterase and butyrylcholinesterase, which may be used in supporting the Alzheimer disease treatment [13].

Mushrooms are the source of easily digestible macro- and microelements due to the natural ability of mycelium to collect and accumulate metals. The metals’ cations in *A. bisporus* mycelium are constituents of many enzymes acting as enzyme activators. The following are the bioelements found in the fruiting bodies of *A. bisporus* in the highest amounts: zinc, phosphorus, magnesium, manganese, copper, potassium, selenium, sulfur, sodium, and calcium [14,15,16,17,18,19]. The health benefits of this mushroom and its ability to accumulate bio-nutrients has made this species a natural component of diet which could supplement macro- and micronutrients deficiencies if diagnosed. The type and content of bioelements in the fruiting bodies of mushrooms is determined by many factors, among others the cultivation conditions (metal content in the substrate), origin of mushroom species, and stage of mycelial growth. Therefore, obtaining the representative research material reflecting its characteristic in the natural environment is difficult. High tolerance of mushroom to heavy metals presence is justified in their specific ability to eliminate reactive oxygen radicals, which is the natural defense mechanism of mycelium [16,18]. This mechanism is based on bonding and “immobilizing” of metal because of rapid intracellular production and extracellular chelating compounds such as thiol functional groups (SH). The remaining and equally important compounds that accompany SH groups for antitoxic reaction are: metallothionein (MT), glutathione (GSH), phytochelatin (PCS), and plastocyanin, which transport excess of metal to mushroom cells thereby reducing their toxic effect in the cell itself [16,18].

Many methods of instrumental analysis are used to determine the content of metals in biological samples—mushrooms. The most important ones include inductively coupled plasma atomic emission spectrometry (ICP-AES) [20], fluorescent total reflection X-ray spectroscopy (TXRF) [21], and atomic absorption spectrometry (ASA) [22]. In most cases, in in vitro cultures, the metal concentration is determined in the obtained biomass. On the other hand, in order to optimize the elemental composition of the in vitro culture, it seems to be an important aspect to monitor the composition of the medium during the growth of the mycelium while maintaining its sterility. The aim of the study is to conduct in vitro cultures of *A. bisporus* on media enriched with the selected macro- and micronutrients in vitro, and to monitor the content of nutrients in liquid medium. This work specifies the reproducibility of biomass growth and bioassay accumulation in biomass (Mg, Zn, Cu, and Fe) for in vitro cultures of *A. bisporus* selected from 81 bases of in vitro cultures derived from substrates enriched with the selected elements in varying proportions. For actual research, the most optimal five types of culture media were selected based on Krakowska et al. (2016) previous experimental conditions. These studies were conducted in order to determine the relationships between biomass growth and macro- and microelement accumulation in *A. bisporus* mycelium, and nutrients’ concentrations in the culture media. The optimum proportions of Mg, Zn, Cu, and Fe were also determined to obtain mycelium rich in these bioelements. Hence, the design of the dipper that would allow the medium to be taken without affecting the biomass growth and its quality was an important aspect of the research. The addition of four biologically active elements in optimum proportions can be used in commercial cultures to obtain a valuable nutritional product.

## 2. Materials and Methods

### 2.1. Mushroom Materials

#### 2.1.1. Initial Mycelial Cultures

Oddoux medium substrates (1957) were weighed to the nearest 0.01 g and the pH was stabilized with 1 M NaOH (pH = 6). Glassware, tools, and media were sterilized for 20 min in a vertical steam sterilizer (ASVE type, 0.1 MPa pressure, 121 °C). All activities related to conduct and passage of in vitro cultures were performed under sterile conditions in a laminar flow box (air flow 0.45 m/s) previously sterilized by UV-C radiation. The in vitro culture of *A. bisporus* was derived from explants that came from the hymenial part of the fruiting bodies. The obtained explants were degreased with 70% ethyl alcohol under 30-s sterilization using sodium hypochlorite, and then rinsed several times with redistilled water. The prepared mycelium fragments, under sterile conditions (under laminar flow), were transferred to Petri dishes, with an Oddoux medium solidified with agar.

#### 2.1.2. Experimental Mycelial Cultures

In the conducted studies, the experimental cultures were derived from the initial cultures obtained on a solid medium. Total of 0.1 g of inoculum was passed to the Erlenmeyer flasks (500 mL) filled with 250 mL of Oddoux liquid medium. After 21 days of culture growth on liquid medium, mycelium were used to set up five experimental cultures enriched with metal salts (CuSO_4_·5H_2_O, FeCl_3_, MgSO_4_·7H_2_O, ZnSO_4_·7H_2_O). The physicochemical conditions of in vitro culture of *A. bisporus* on Oddoux-modified liquid media were established based on previous studies [23,24]. In vitro culture was performed in a medium with pH of 6.0 under conditions simulating a natural day-night photoperiod (12-h light exposure—900 lux/12 h without light) at 25 ± 2 °C.

Both the in vitro culture conditions and the metals’ additives to media (Mg, Zn, Cu, and Fe) were developed as for in vitro cultures of *A. bisporus* obtained by Krakowska et al. (Table 1) in the previous research (Krakowska et al., 2016).

The experiment was designed to confirm the reproducibility of growth and composition (metal content) in the selected optimal media variants (Table 2).

In vitro cultures were then established in five independent replications of the corresponding metal addition variant, and were designed to determine the composition of the medium by monitoring it using two sampling methods: indirect and direct one. During the 21-day growth of mycelium, 2 mL of media were collected daily using direct and indirect systems. The biomass obtained after the culture was separated from the liquid medium using Büchner filter paper and funnel, washed four times with distilled water, and then lyophilized (Labconco 4.5, temperature 40 °C).

### 2.2. Reagents

The chemicals of analytical purity such as CuSO_4_·5H_2_O, FeCl_3_, MgSO_4_·7H_2_O, and ZnSO_4_·7H_2_O were purchased from POCH (Gliwice, Poland). HNO_3_ (65%) and H_2_O_2_ (30%) Suprapure^®^ were purchased from Merck (Darmstadt, Germany). Four times distilled water filtered through Millipore Millex GP—0.22 μm was applied to prepare culture media. For sterilization of initial in vitro culture, 70% ethanol (Merck, Darmstadt) and 15% sodium hypochlorite from Unilever (Unilever, Hungary) were used. The UHU^®^ epoxy resin manufactured by GmbH & Co in Germany was used to fill the test probe chamber.

### 2.3. Direct Sampling Methods of Medium from In Vitro Cultures

Simpler variant of a system for the direct sampling of liquid medium for chemical analysis relied in assembling a valve at the port to the bottom of the Erlenmeyer flask with in vitro culture of *A. bisporus*. A set of 10 flasks of 500 mL volume was modified by blending into their side walls, at a height of about 1.5–2 cm above the bottom, glass tubes with inner diameter of 4 mm, and length of 2 cm. The drain plugs are equipped with sterile gastric tube plugs to act as a hinged valve. The plugs slid onto the glass tube were sealed with heat-shrink shirts.

### 2.4. Indirect Sampling Methods from Medium from In Vitro Cultures by Copyright Constructed Dipper

In the indirect composition monitoring system of liquid media, a cylindrical probe (dipper) was immersed in a 500 mL Erlenmeyer flask containing a semi-permeable membrane and a Teflon exit capillary through which a sample of the “receiving” solution for analysis was taken by using a sterile syringe. Sterile diaphragm probes—immersion probes were filled with sterile, four times distilled water before immersing in the liquid media. The probes were made to use semipermeable membranes that allowed Mg, Zn, Cu, and Fe ions diffusion according to the chemical potential gradient from the higher concentration solution (liquid medium) to the lower concentration solution (distilled water), that filled the interior of the probe.

**Figure 1:**
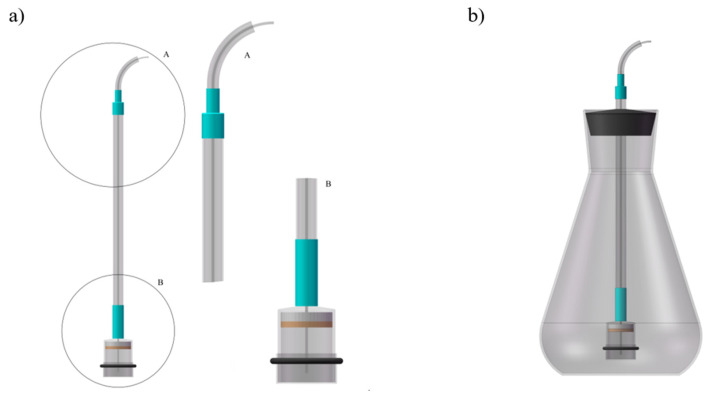
(**a**) Construction of the test probe, (**b**) probe in a mycelium in in vitro culture of *A. bisporus* flask (A—probe shaft, B—dipper).

Figure 1 illustrates the method of the probe manufacturing. A 4 mL blown chamber was cut into two equal parts of 2 mL each. Parts of the hydrophobic filter compartment were fitted with a flexible tube made of Tygon^®^, onto which a roller clamp was then inserted which acted as a control valve. A Teflon capillary of 1 mm internal diameter was inserted into the “light” of the flexible tubing, one of its ends was approximately 2 mm from the surface of the membrane and the other protrudes from the tubing at least 2 cm. The purpose of the stiffening of the capillary tube construction was to remove a glass tube, and using heat-shrinkable tubing, it was connected to the drip chamber in order to seal the capillary. Teflon joint, inside of the drip chamber, was filled with a 3 mm layer of liquid epoxy resin. On the cutting side, a disk from the semi-permeable membrane was attached to the chamber, which was attached using an O-ring (Figure 1a). The probe was rinsed in methanol, UV sterilized, and then filled with sterile, distilled water. Such probes were immediately mounted in flasks (Figure 1b), under sterile conditions and under laminar flow. Before the study, a number of experiments were conducted, allowing us to determine the minimum time after which the steady state of the metal concentrations on both sides of the membrane was determined, if one of the solutions was on one side and the other on the other side. The first sampling time after placement of the probe was set at 4 h, and then every 24 h.

### 2.5. Determination of Elements with the Atomic Absorption Spectrometry (AAS)

The resulting lyophilized biomass was homogenized in an agar mortar, and then mineralized in a closed wet system. Therefore, 0.2 g was weighed and transferred into Teflon vessels. Mineralization was conducted using a microwave system (Anton Paar Multiwave 3000, Buchs, Switzerland) using 8 mL of concentrated nitric acid(V) and 2 mL of hydrogen peroxide. The mineralization solution was transferred to a quartz crucible and excess of reagents was evaporated at 80 °C for 50 min. The resulting sample was quantitatively transferred into 10mL flasks. All samples were digested in three independent replicates. The obtained analytical samples were analyzed for the presence of Mg, Zn, Cu, and Fe by means of flame atomic absorption spectrometry (F-AAS) technique in a Perkin-Elmer Model 3110 (USA) spectrometer. Table 3 presents the results for repeatability of the selected optimal in vitro cultures of *A. bisporus*.

**Figure 2:**
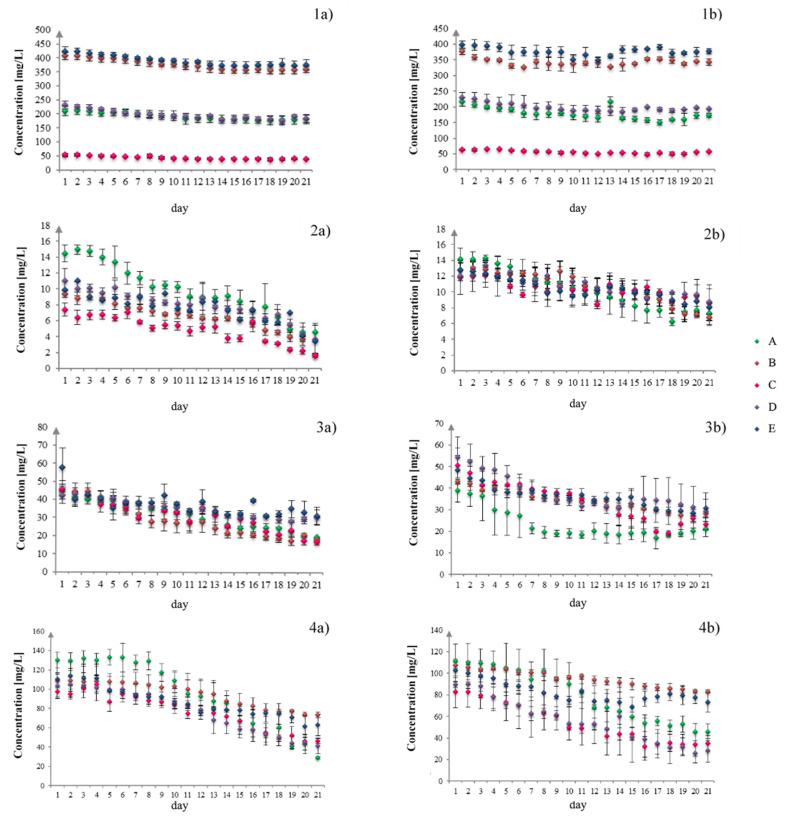
Changes in Mg(1), Zn(2), Cu(3), and Fe(4) concentration in *A. bisporus* in vitro culture medium as a function of time; (**a**) indirect sampling and (**b**) direct sampling (A, B, C, D, E—symbol identify in vitro culture—according to Table 2).

Figure 2 presents the results of F-AAS determinations of metals’ concentrations (Mg, Zn, Cu, and Fe) in liquid media during the growth of mycelium.

**Figure 3:**
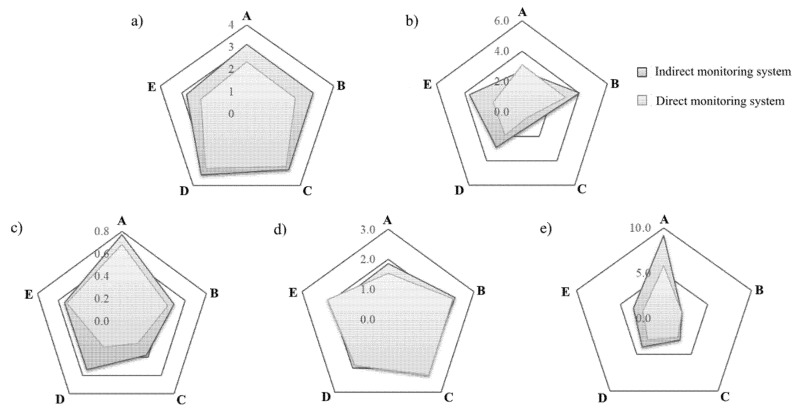
Radar graph showing (**a**) the distribution of biomass [g] growth in *A. bisporus* in vitro cultures, and the accumulation of (**b**) Mg, (**c**) Zn, (**d**) Cu, (**e**) Fe [mg/g] in biomass obtained from *A. bisporus* in vitro cultures depending on the system of monitoring the composition of the medium (A, B, C, D, E—symbol identify in vitro culture—according to Table 2).

Figure 3 shows the degree of bioaccumulation of the metals and biomass growth in in vitro cultures using direct and indirect media of nutrient composition monitoring.

## 3. Results and Discussion

### 3.1. Evaluation of the Reproducibility of Optimal A. bisporus In Vitro Cultures

The experiments conducted on liquid in vitro cultures of *A. bisporus*, on the modified media selected as optimal, demonstrated that differences in average biomass growth did not exceed 5% (Table 3). A similar trend was observed by comparing average biomass growth (differences not greater than 5%) of the same in vitro cultures to the previous results obtained in Krakowska et al.’s (2016) work and the selected in vitro optimal cultures studied in the present study (Table 3). Significant differences were found by comparing the mean content of Mg, Zn, Cu, and Fe in *A. bisporus* mycelium (Table 3) with the results obtained for the same in vitro culture in Krakowska et al.’s (2016) research. Mg ranged from 2% (in vitro culture A) to 36% (in vitro culture C), Zn from 14% (in vitro culture E) to 21% (in vitro culture C), Fe from 4.1% (in vitro culture B) to 26% (in vitro culture B), and for Cu from 12.7% (in vitro culture C) to 28.1% (in vitro culture E). Relatively small differences in biomass growth (<5%) were observed between each in vitro cultures, and significantly higher (≤28%) differences were observed when comparing the individual metals in biomass. This can be considered acceptable and satisfying in very high variability of the parameters in biological material. It was concluded that cultures of *A. bisporus* media with modified chemical composition (Table 2) are reproducible with respect to biomass growth and bio-accumulation efficiency of Mg, Zn, Cu, and Fe.

### 3.2. Evaluation of Biomass Growth and Metals’ Accumulation Efficiency in Mycelium from In Vitro Cultures of A. bisporus

The results of average biomass growth for *A. bisporus* in vitro cultures performed on the modified liquid media differed significantly depending on the method of sample extraction for F-AAS analysis of Mg, Zn, Cu, and Fe concentration in the medium (Figure 3a). Average biomass increments for in vitro cultures monitored by the probe were 24.94% (in vitro culture A), 27.02% (in vitro culture B), 5.89% (in vitro culture C), 9.96% (in vitro culture D), and 23.25% (in vitro culture E) respectively, and were higher than in analogous cultures, from which the samples were collected directly. The biomass increases obtained in the study for in vitro cultures of the species *A. bisporus* carried out on media enriched with bioelements (even the lowest—9.986 g/L of Oddoux liquid medium) were higher than the increases in biomass obtained for both white and brown champignon by Rashid et al.’s (2018) where the culture media was enriched with extracts from Sesbania sesban straw and phosphate rock (4.24 g/L) [25]. Moreover, other scientists Tan et al. (1992) and Hassan et al. (2012) conducted research on the increase in the biomass of in vitro cultures of other mushroom species, including the popular in Europe—*Lentinula edodes* or *Flammulina velutipes* (5.65 g/L medium) obtained lower biomass growth [26,27]. The efficiency of Mg, Zn, Cu, and Fe accumulation in biomass from in vitro cultures of *A. bisporus* indirectly monitored by the test probe, and direct sampling of media was also assessed just as the growth of biomass (Figure 2). Similar to the biomass growth factors, efficiency of bioaccumulation of metals was significantly higher for in vitro cultures monitored by means of the dipper.

In vitro cultures of *A. bisporus* on the modified liquid media in which Mg, Zn, Cu, and Fe concentrations were indirectly monitored by the test probe showed significantly higher biomass and metals’ accumulation ratios, than the cultures from which the media sample was taken directly. By direct sampling of the medium through the glass jug using a sterile syringe, in addition to the nutrient solution, small amounts of mycelium were also taken in. Initially, the amount of mycelium accumulated in the medium was small, but over time it rapidly increased, which significantly impedes the uptake of the nutrient solution without fragments of the mycelium. Nevertheless, taking even small amounts of mycelium right at the beginning of culture should also result in a decrease of its growth. Successful growth of in vitro culture of *A. bisporus* on liquid nutrient media modified with relatively high addition of Mg, Zn, Cu, and Fe supports the use of an indirect system for the samples collection using a test probe, which would only slightly disturb the natural cycle of the growth of mycelium. Direct sampling methods of liquid media from the in vitro culture vessel requires special caution because of the potential for infection of cultures (in vitro culture C) and leads to significant reductions in biomarker growth and F-AAS analysis errors due to complex matrix effects.

### 3.3. Evaluation of the Changes in Chemical Composition of Liquid Media during Biomass Growth Using Indirect and Direct Sampling Methods

Changes of the media composition during the growth of *A. bisporus* mycelium were characterized. Average changes in metals’ concentrations (Mg, Zn, Cu, and Fe) indicate significant regularities. For in vitro cultures of *A. bisporus*, whether the sampling was conducted directly or indirectly, an increased periodic accumulation of Mg, Zn, and Cu in mycelium was observed. The maximum periods of this increased metal uptake were: 1–13 days for Mg, 1–16 days for Zn, and 1–15 days for Cu. By analyzing temporal changes in Fe concentration, the reverse trend was observed.

In the initial phase of mycelium growth, the concentration of Fe in the medium was stable, apparent decrease of its concentration was observed only between 9 and 14 days of culture. Periodic changes in concentrations of Mg, Zn, and Cu are justified during mycelial growth curve (Muszyńska et al., 2019) [28]. In the first phase of mycelium growth, Mg, Zn, and Cu are taken from the liquid medium until the growth of mycelium is stopped, which occurs about 14 days after the in vitro culture is established. This is obvious because bio-nutrients such as Mg, Zn, and Cu are essential for the proper growth of mycelium. No further changes in metal concentrations were observed starting from the 14th day of the culture. The concentrations of Mg, Zn, and Cu in the medium solution confirms that they no longer accumulate in the growing mycelium (Figure 2). Iron is necessary in very small amounts for the development and growth of mycelium, too high iron concentration may lead to apoptosis of fungal cells, therefore the applied supplement to the media was small (Table 1). The best results for biomass growth and metal accumulation were obtained for in vitro cultures of *A. bisporus*, grown on the medium with the lowest iron supplement, namely, 0.1 g Fe per 250 mL of medium. In addition, in the media with higher Fe concentrations, the inhibitory effect on biomass growth was observed.

## 4. Conclusions

The success of the present work was owing to the construction of a dipper which allowed for monitoring of the changes in Mg, Zn, Cu, and Fe concentrations in liquid media during biomass growth. Hence, this method, in which the probe was used, did not affect the culturing conditions; hence, the highest biomass growth and metal accumulation rates were obtained. Direct sampling method, although very simple in implementation, violated the conditions of mycelial growth, resulting in lower biomass and metal accumulation, as well as cases of infection of cultures. Spectroscopic analysis of the changes in metal concentration over time allowed to correlate well the results obtained in individual stages of mycelial of metal addition to the *A. bisporus* culture medium. This confirms the hypothesis that the mycelium spontaneously regulates the type and quantity of metals taken from the environment in a way that maximizes its growth. Among the two methods of sampling from in vitro cultures used in the study, the indirect method can be successfully used in the future to monitor changes in the composition of liquid media for in vitro culture in order to obtain mycelium with a specific optimized composition, both qualitative and quantitative.

## Figures and Tables

**Table 1:** Amount of macro- and micronutrients added to the medium used for in vitro cultures of *A. bisporus*.

Metal	Amount of Additive [g] per 250 mL Oddoux Liquid Medium			
	0	1	2	3
Mg	0	0.10	0.50	1.00
Zn	0	0.01	0.05	0.10
Cu	0	0.01	0.05	0.10
Fe	0	0.10	0.50	1.00

**Table 2:** Amounts of metal additives in the medium selected for optimal in vitro culture of *A. bisporus*.

Symbol Identify In Vitro Culture	The Proportions of Added Metals [g]
A	Mg(2)Zn(1)Cu(2)Fe(1)
B	Mg(3)Zn(1)Cu(2)Fe(1)
C	Mg(1)Zn(1)Cu(3)Fe(1)
D	Mg(2)Zn(1)Cu(3)Fe(1)
E	Mg(3)Zn(1)Cu(3)Fe(1)

**Table 3:** Results of measurement of biomass growth and metal accumulation in five selected in vitro cultures of *A. bisporus*.

In Vitro Culture	Repetition	Weight [g]	Concentration [mg/g d. w.]			
			Mg	Zn	Cu	Fe
Control		2.1263	0.91 ± 0.06	0.042 ± 0.004	0.011 ± 0.004	1.02 ± 0.08
A	A.1	3.3309	2.91 ± 0.01 *	0.899 ± 0.001 *	1.598 ± 0.008 *	9.30 ± 0.01 *
	A.2	3.3229	2.06 ± 0.01 *	0.650 ± 0.006 *	1.942 ± 0.003 *	8.97 ± 0.01 *
	A.3	3.1996	2.90 ± 0.01 *	0.705 ± 0.003 *	2.175 ± 0.009 *	8.43 ± 0.02 *
	A.4	3.2827	3.32 ± 0.03 *	0.716 ± 0.011 *	1.693 ± 0.007 *	7.80 ± 0.01 *
	A.5	3.0178	2.78 ± 0.01 *	0.576 ± 0.004 *	2.234 ± 0.002 *	7.42 ± 0.02 *
B	B.1	3.1333	4.63 ± 0.02 *	0.542 ± 0.006 *	2.278 ± 0.001 *	2.61 ± 0.02 *
	B.2	3.2668	5.15 ± 0.01 *	0.641 ± 0.009 *	2.601 ± 0.011 *	3.63 ± 0.01 *
	B.3	3.2095	4.10 ± 0.02 *	0.677 ± 0.002 *	3.141 ± 0.007 *	2.16 ± 0.03 *
	B.4	3.1925	4.49 ± 0.02 *	0.581 ± 0.005 *	2.523 ± 0.008 *	3.02 ± 0.01 *
	B.5	2.9832	4.42 ± 0.01 *	0.488 ± 0.006 *	2.994 ± 0.002 *	2.12 ± 0.02 *
C	C.1	3.3093	1.15 ± 0.02 *	0.381 ± 0.009 *	2.477 ± 0.012 *	3.64 ± 0.03 *
	C.2	3.0408	1.01 ± 0.01	0.168 ± 0.010 *	2.377 ± 0.015 *	1.37 ± 0.02 *
	C.3	3.4291	1.83 ± 0.01 *	0.483 ± 0.007 *	1.999 ± 0.028 *	3.59 ± 0.03 *
	C.4	3.2193	0.90 ± 0.03	0.290 ± 0.001 *	3.375 ± 0.001 *	4.02 ± 0.02 *
	C.5	3.5032	1.33 ± 0.01 *	0.462 ± 0.004 *	3.455 ± 0.018 *	4.80 ± 0.04 *
D	D.1	3.2917	2.25 ± 0.03 *	0.821 ± 0.009 *	3.111 ± 0.022 *	6.91 ± 0.03 *
	D.2	3.3175	2.92 ± 0.01 *	0.711 ± 0.002 *	1.830 ± 0.010 *	4.28 ± 0.03 *
	D.3	3.2144	2.70 ± 0.03 *	0.655 ± 0.001 *	2.107 ± 0.002 *	5.44 ± 0.01 *
	D.4	3.8956	2.83 ± 0.01 *	0.661 ± 0.008 *	2.133 ± 0.021 *	3.99 ± 0.03 *
	D.5	2.4965	2.17 ± 0.01 *	0.581 ± 0.010 *	1.597 ± 0.001 *	5.59 ± 0.03 *
E	E.1	2.6613	4.59 ± 0.04 *	0.607 ± 0.009 *	2.565 ± 0.008 *	3.62 ± 0.02 *
	E.2	2.9828	5.13 ± 0.03 *	0.564 ± 0.002 *	2.873 ± 0.009 *	3.77 ± 0.02 *
	E.3	2.7405	5.00 ± 0.01 *	0.713 ± 0.006 *	3.462 ± 0.002 *	2.75 ± 0.01 *
	E.4	2.7925	3.71 ± 0.03 *	0.647 ± 0.005 *	1.699 ± 0.012 *	3.01 ± 0.03 *
	E.5	2.3925	3.77 ± 0.01 *	0.665 ± 0.011 *	1.974 ± 0.011 *	3.96 ± 0.02 *

* Data were presented as the mean ± SD; *n* = 3 in triplicate; one-way ANOVA with post hoc Tukey test; * *p* < 0.0001 vs. Control (GraphPad Prism 8.0).

## Data Availability

Data is contained within the article.

## Sample Availability

Samples of the compounds are not available from the authors.
